# The Real Cost of “Cosmetic Tourism” Cost Analysis Study of “Cosmetic Tourism” Complications Presenting to a Public Hospital

**Published:** 2015-07-28

**Authors:** Ryan Livingston, Paul Berlund, Jade Eccles-Smith, Raja Sawhney

**Affiliations:** ^a^University of Queensland, Plastic & Reconstructive Surgery Registrar Gold Coast University Hospital, Queensland, Australia; ^b^Gold Coast University Hospital, Queensland, Australia; ^c^Bond University, Queensland, Australia; ^d^Plastic & Reconstructive Surgery Gold Coast University Hospital, Queensland, Australia

**Keywords:** cosmetic tourism, cost analysis, operations abroad, complications, medical tourism

## Abstract

“Cosmetic Tourism,” the process of traveling overseas for cosmetic procedures, is an expanding global phenomenon. The model of care by which these services are delivered can limit perioperative assessment and postoperative follow-up. Our aim was to establish the number and type of complications being treated by a secondary referral hospital resulting from “cosmetic tourism” and the cost that has been incurred by the hospital in a 1-year period. Retrospective cost analysis and chart review of patients admitted to the hospital between the financial year of 2012 and 2013 were performed. Twelve “cosmetic tourism” patients presented to the hospital, requiring admission during the study period. Breast augmentation was the most common procedure and infected prosthesis was the most common complication (n = 4). Complications ranged from infection, pulmonary embolism to penile necrosis. The average cost of treating these patients was $AUD 12 597.71. The overall financial burden of the complication to the hospital was AUD$151 172.52. The “cosmetic tourism” model of care appears to be, in some cases, suboptimal for patients and their regional hospitals. In the cases presented in this study, it appears that care falls on the patient local hospital and home country to deal with the complications from their surgery abroad. This incurs a financial cost to that hospital in addition to redirecting medical resources that would otherwise be utilized for treating noncosmetic complications, without any remuneration to the local provider.

Cosmetic tourism, the process of traveling overseas for cosmetic procedures, appears to be an expanding global phenomenon.^1^ The general public's perception of cosmetic tourism is changing, with growing numbers considering traveling overseas for cosmetic procedures. It appears in part because of the lower cost of surgery as well as the increased incidence of global travel and low-cost airfares.^1^ Despite this, there is a paucity of data and discussion surrounding the incidence and management of complications, and the current model of care used by these international providers. While the number of patients who are traveling for procedures is currently unknown, media reports claim that up to 15 000 Australian women are traveling overseas for cosmetic procedures each year.^2^ A basic Internet search reveals an entire industry, which promotes cosmetic surgery at reduced prices. Recent reports on mainstream current affair programs have been highlighting this emerging trend, increasingly bringing cosmetic tourism into the limelight.^3^

The model of care by which these services are delivered limits preoperative assessment and follow-up to a few days to a week. As a result, complications due to these procedures tend to present after the patient has returned from his or her “holiday.” Complications from these surgeries are not uncommon. In one study, 16.5% of patients experienced complications, with 8.7% receiving further treatment in the publicly funded health system on return home.^4^ There are reports that an increasing number of patients with complications from such procedures are presenting to public hospitals.^5^

## METHODS

The aim of the study was to establish the number and type of complications being treated by a surgical unit at a secondary referral hospital. We also planned to perform a cost analysis of treating such patients and determine the following: nature of the treatment; duration of hospital admission; need for repeat surgical procedures; and follow-up.

We performed a retrospective analysis of patients presenting between the financial year of June 2012 and June 2013 at the Gold Coast Hospital. The patient population was identified using the *ICD-10* AM (Australian modification) codes for complications of surgery by the hospital case mix reporting service. Using a standardized pro forma, a chart review was performed noting the patients' demographics, location of surgery, type of surgery, the complication that occurred, and the treatment required. With regard to treatment, we noted the need for hospital admission and duration of stay, the number of surgeries required, the intravenous use of antibiotic drugs and duration, as well as the number of follow-up outpatient appointments attended.

The patient's unique reference number was provided to the activity-based costing team. Using the clinical costing system (sunrise decision support manager), patient-level costs were calculated for each patient.

## RESULTS

During the 1-year study period, 12 patients with “cosmetic tourism” complications who presented to the emergency department were admitted to our hospital. All of the patients had their operations performed in Thailand. Breast augmentation was the most common procedure (*n* = 10). Four patients had multiple procedures (Table 1). In 2 cases, it was documented that the patients had undergone dental procedures shortly after cosmetic surgery. It was not indicated whether the remaining patients in the study underwent dental treatment. Three patients were smokers, smoking not only through the perioperative period but also through the postoperative recovery period.

**Figure 1:**
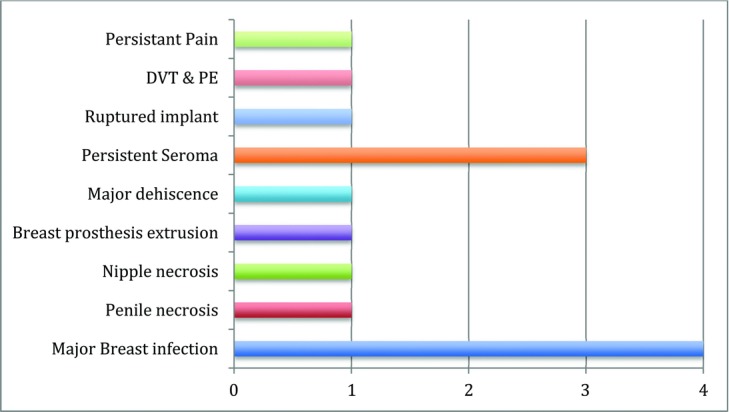
Number and type of cosmetic complications.

**Figure 2:**
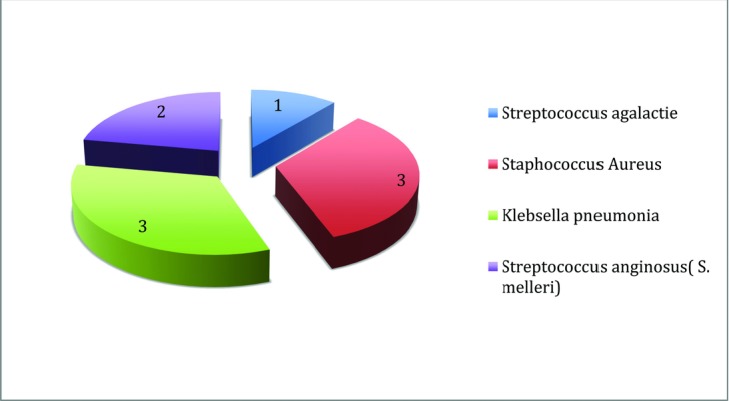
Organisms isolated in infected cases.

The complications treated were varied, ranging from nipple or penile necrosis to pulmonary embolism (Fig 1). The most common complication was infected implants after breast augmentation (*n* = 4). The infective organisms found were mainly streptococci and staphylococci species (Fig 2). A fungus was isolated in 1 patient. Multiresistant organisms were not common (*n* = 1).

Inpatient admission averaged 6 days per complication with a range of 0 to 15 days. The cohort had 67 inpatient days in total. On average, each patient had 1 operation (range, 0–5), and as a group 12 operations were performed. Two of the patients had documentation, indicating private referral for ongoing care and surgery. Out of the patients who did not seek private referral, 4 have not finished treatment and are still requiring further management or surgery at the public hospital. On average, each required 5 outpatient reviews (range, 0-9).

Cosmetic tourism complications presenting to this hospital in this study have a reported cost of AUD$151 172.52. The most spent on a single patient was $AUD 33 060.02 and average amount was $AUD12 597.71 (Table 2).

## DISCUSSION

Our study has demonstrated a range of complications that have occurred as a result of cosmetic surgery performed overseas. While our study focused on cosmetic treatments, the authors believe that other specialties will begin to see an influx of complications from other procedures such as in vitro fertilization, arthroplasty, and stem cell treatments.

The patients treated in our department had acute complications that had the potential for significant morbidity. Some people may argue that since these complications are the result of elective cosmetic surgery performed in a different country, any complications are an unnecessary burden on the health service. Introducing legislation to ensure that all these patients have compulsory medical insurance would be one potential method of recouping money spent on these patients' complications.

Minimizing complications is essential and requires more than just surgical skill; appropriate preoperative assessment and postoperative follow-up by the physician performing the procedure. Our aim is not to criticize the surgical skill of our overseas colleagues; however, analyzing the model of care by which the service is being delivered is worthwhile. Perioperative counseling could be deemed to be inadequate by our national standards and there are unconfirmed reports from our patients that they were seen together in groups. Postoperative follow-up is limited to the short period of time the patient spends in his or her holiday location.

The American Society of Plastic Surgeons outlines many of these concerns in a briefing paper, including the increased risk of having treatment overseas. The Society also suggests that patients treat the perioperative time as a holiday and this can negatively impact a patient's healing process. Examples given in the briefing are increased smoking and alcohol consumption, excessive sunbathing, swimming, and walking tours in addition to other tourist activities.^6^ The risk of long haul flights pre- and postsurgery is also of concern. Pulmonary embolism was a complication found in 1 of our study population returning from Thailand.

Australian practitioners abide by stringent and heavily regulated guidelines. The standard of care provided by international providers may be different not only in pre- and postoperative care, but also in regard to products, equipment, nursing staff, and medical training. This is not something that can be regulated by Australia, as there are no systems in place that the authors are aware of to do so. As such, quality of service outside of Australia would be impossible to guarantee. Legal recourse for a complication from overseas surgery would be arduous if not impossible even in cases of gross medical negligence.^7^ The introduction of “joint commission international” accreditation scheme in more recent years takes a step to relieve some of these misgivings.^8^

Furthermore, the staffing ratio of doctors to patients in Thailand is less compared with that of Australia. This may not just have ramifications for the Australian tourist but also for the local Thai population that loses the skills of a locally practicing doctor that now participates in medical tourism. On the “flip side,” it may be that the money from these types of enterprises is of benefit to the foreign health system and economy and as a consequence better medical equipment and services are a resource that the local populace can draw on.

The loss to the Australian economy from private medical tourism is obvious. If looking at Thailand alone, 1.5 million foreigners were treated in their hospitals in 2009, making their economy US$6 billion.^8^

For a patient opting to source procedures overseas, their initial contact in Australia is often a broker, who locates a doctor to perform specific treatment, as well as organizing flights and accommodation. The patient may have made a significant commitment toward having the procedure even before they have a consultation with a doctor. This type of setup has the potential to make it difficult for patients to withdraw even if they have second thoughts about the intervention.

A concern for the authors was the number of patients who underwent dental treatment during the postoperative period. The performance of dental procedures was clearly documented in 2 patients’ notes and may have occurred in others, but as there was a lack of documentation, it made it difficult to assess. Dental procedures add an additional risk for infective complications to implanted prostheses.^9^^-^11 Ongoing cigarette use was documented in 3 of the patients’ charts. In our health service, these patients who continued to smoke would have been likely to be refused the surgery they received, because of the significant increased risk of postoperative complications.^12^

Multiresistant organisms in Asia such as multiresistant *Staphylococcus aureus* have a higher prevalence than those in Australia. Some Asian countries have a prevalence bordering on 70%.^8^ In our study population, 1 multiresistant organism was cultured.

Unfortunately, the exact numbers of people receiving cosmetic surgery abroad are not known to the authors; neither are the numbers of Australians who return from cosmetic holidays with complications. As such, it is not possible to accurately compare local and “cosmetic tourism” complication rates. This would be a useful comparison to further evaluate whether this model of care incurred an additional increased risk to the patient.

## CONCLUSION

The financial burden of cosmetic tourism to our hospital over a 1-year period was AUD$151 172.52. This figure, of course, cannot account for the emotional or psychological cost to patients whose surgical experience ends with significant complications or morbidity. In conclusion, this study has demonstrated a range of complications as a result of patients engaging in “cosmetic tourism.” We have shown that there is a financial burden being incurred from these complications. These findings support the need for increased public health strategies in the aims of prevention of morbidity and mortality and the future management and education of patients engaging in “cosmetic tourism.”

## Figures and Tables

**Table 1:** Cosmetic procedures performed abroad

Procedures	No. of procedures
Breast augmentation	10
Labiaplasty	1
Penile augment	1
Chin lift	1
Mastopexy	3
Abdominoplasty	1
Brachioplasty	1
Liposuction	1
Multiple procedures (nonbreast)	4

**Table 2:** Treatment cost per patient

Patient number	Cost, $AUD
1	9 812.64
2	1 190.28
3	14 753.68
4	2 695.92
5	1 819.06
6	10 667.91
7	8 168.67
8	4 472.44
9	23 029.60
10	33 060.02
11	24 186.83
12	17 315.47
Total	151 172.52

## References

[1] Karuppan CM, Karuppan M (2010). Changing trends in health care tourism. Health Care Manage.

[2] ABC Radio Australia Thousands of Aussies head overseas for plastic surgery. http://www.radioaustralia.net.au/international/radio/program/connect-asia/thousands-of-aussies-head-overseas-for-plastic-surgery/1055224.

[3] Illawarra Mercury Newspaper “My breasts are perfect”: overseas surgery debate. http://www.illawarramercury.com.au/story/2184393/my-breasts-are-perfect-overseas-surgery-debate.

[4] Centre for Interdisciplinary Gender Studies Sun, Sea, Sand and Silicone: Aesthetic Surgery Tourism.

[5] Menlendez MM, Alizadeh X (2011). Complications from international surgery tourism. Aesthet Surg J.

[6] American Society of Plastic Surgeons Dangers of Plastic Surgery Tourism.

[7] Clark P, Adegunsoye A, Capuzzi K, Gatta D (2013). Medical tourism: winners and losers. Internet J Health.

[8] Department of Resource Energy and Tourism Medical Tourism in Australia: A Scoping Study.

[9] Brand KG (1993). Infection of mammary prostheses: a survey and the question of prevention. Ann Plast Surg.

[10] Hunter J, Padilla M, Cooper-Vastola S (1996). Late *Clostridium perfringens* breast implant infection after dental treatment. Ann Plast Surg.

[11] Chang J, Lee G (2011). Late hematogenous bacterial infections of breast implants: two case reports of unique bacterial infections. Ann Plast Surg.

[12] Coon D, Tuffaha S, Christensen J, Bonawitz SC (2013). Plastic surgery and smoking: a prospective analysis of incidence, compliance and complications. Plast Reconst Surg.

